# “This is your brain on drugs”: adolescent substance use prevention through neuroscience education

**DOI:** 10.1186/1940-0640-8-S1-A32

**Published:** 2013-09-04

**Authors:** Sion K Harris, Stephanie Doyle, Lon Sherritt, Shari Van Hook, John R Knight

**Affiliations:** 1 Boston Children’s Hospital, Boston, MA, USA; 2 Harvard Medical School, Boston, MA, USA; 3 Boston Public Health Commission, Boston, MA, USA

## 

Interest in the brain has risen dramatically in recent decades, suggesting that neuroscience-based information about substance use risks could be a compelling addition to prevention efforts. However, no studies have tested this approach among adolescents. In this study, we tested the effects of a high school science curriculum called “The Brain: Understanding Neurobiology through the Study of Addiction” developed by the U.S. National Institutes of Health (NIH) to teach students about how alcohol and drugs affect the brain. We examined the curriculum’s effects on students’ knowledge, attitudes, and use of tobacco, alcohol, and drugs. Participants were 9^th^-11^th^ graders attending 2 urban high schools whose parents consented (222/282=79%). We standardized the 5-lesson curriculum and trained 5 science teachers; 8 classes (n=133) received the NIH curriculum, 5 matched-grade comparison classes (n=89) received the usual curriculum. We used a self-administered survey to assess demographics, knowledge, perceived risk of harm from substance use, substance use, peer/family substance use, and prior health education. We collected data 1-2 weeks pre-intervention (T1), immediately post-intervention (T2; 1 month after T1), and 6-8 months later (T3). We used multiple logistic regression with generalized estimating equations for post-test comparisons, adjusting for T1 differences and within-class correlation. 180/222 (81%) students completed all measurements; group completion rates were similar (control=80%, intervention=82%). Compared to control students, intervention students had lower past-30-day cigarette use at T2 (7% vs. 21%, AOR=0.20, 95% CI 0.08-0.49, p=.01), and a marginal trend toward lower alcohol *initiation* between T1 and T2 (3% vs. 20%; adjusted odds ratio [AOR]=0.09 95% CI 0.01-1.03, p=.052). Among low-frequency marijuana users (1-5 times lifetime at baseline), fewer intervention students had past-30-day marijuana use at T2 (5% vs. 35%, AOR=0.01, 95%CI 0.00-0.30, p=0.03). All between-groups differences were extinguished by T3. Neuroscience education is a promising supplemental strategy for adolescent substance use prevention; however, ongoing reinforcement is needed.

